# A Course of Clinical Lectures, Delivered at the Hôtel-Dieu, Paris, for the Session 1842-’43

**Published:** 1843-04-15

**Authors:** A. F. Chomel

**Affiliations:** Paris


### THE MEDICAL EXAMINER,

### And Retrospect of the Medical Sciences.

Vol. VI.] PHILADELPHIA, SATURDAY, APRIL 15, 1843. [No. 7.

### A COURSE OF CLINICAL LECTURES,

*Delivered at the Hôtel Dieu, Paris, for the Session* 1842–'43.

BY A. F. CHOMEL, M. D.

### LECTURE V.

*Severe Double Pneumonia, complicated with Enteritis—Recovery. Remarks on some points in the diagnosis of Pneumonia.*

At No. 10 of the Salle Saint-Bernard, is a woman with an affection which, from the commencement, has appeared to me to be of the gravest nature. This woman is sixty years old; her physiognomy offers a great change; her cheeks are sunken; she lies perpetually on her back; her respiration is frequent and hurried; her skin is hot; her pulse quick; and there is a constant, painful cough, with viscous, yellow, semitransparent expectoration, adherent to the vessel. These phenomena led us to suspect immediately the existence of an acute inflammation of the thoracic organs. Questioned succinctly, (for her extreme state of prostration did not permit us to enter into any long details,) she informed us that when twenty years old she had a catarrhal affection, for which she was largely bled several times. Forty years have elapsed, during which time she has enjoyed good health. About five or six days ago she commenced coughing; early in the morning she had a violent chill, which continued more or less for three days. This was succeeded by abundant sweats when she was in bed, which were followed again by chills, as soon as she exposed herself to the air. On inquiring of this patient whether she had been suddenly cooled before her attack, she answered in the negative; she told us that she did not feel cold, either the evening previous to the day of invasion, or the preceding days; and she added, that she had always led a regular life, and had never been exposed to atmospherical vicissitudes. You will see presently the value of these points of information.

The chill in this patient continued, with a general sensation of cold, for nearly three days; and then the local phenomena were developed one after another. The third day she experienced a severe pain under the right nipple, and an intense diarrhœa supervened, with four or five discharges a day; very fetid matter being rendered each time. A constant distressing cough declared itself at the same time, with viscous, adherent sputa. On the 27th of November she was carried into the hospital. On her entrance she was immediately bled.

On our visit the following morning her condition was as follows: Great dejection; general feebleness; anxiety; face injected; skin dry and hot; pulse 110; respiration oppressed and hurried, (40 per minute;) expectoration viscid and adherent; mouth dry, as well as the throat; belly slightly meteoric, (the diarrhœa had ceased since the evening;) insomnia for five days; entire want of appetite. On ausculting her, we found posteriorly, at the summit, and at the two sides, a dry, well marked bronchitic souffle over a space as large as the palm of the hand. When the patient was made to cough, some humid rales were heard, but no genuine crepitation. Under the axilla there were a few bubbles, and here and there a little mucous rale in the bronchi, at the posterior and inferior part of the chest. Under the left nipple, the seat of pain, you heard a pure respiratory murmur. Thus we had a severe pneumonia to treat, especially grave from the phenomena which accompanied it.

What was the appropriate treatment in such a case? Sanguine evacuations seemed to me hazardous, on account of the great feebleness of the patient; it was rather a case for the use of tartar emetic; but there existed considerable intestinal irritation, which might be aggravated by the antimonial. Hence it was thought advisable to postpone its employment, at least for the time, and to avail ourselves of it at a later period, should it be deemed advisable. We relied for the time on blisters, whose great utility in such cases has been largely demonstrated by my experience. A large one was accordingly applied under the axilla; that region being preferable to the back, on account of the inconvenience and pain they produce there, especially in patients who already suffer much, and are weakened by a disease of some duration. It may also act advantageously on the intestinal inflammation, by making a favourable revulsion. To this treatment were added enemata and emollient drinks. On the third day the state of the patient somewhat improved; the diarrhœa entirely ceased; there was but little pain on pressure; and it remained only slightly meteoric. The cessation of the abdominal symptoms we considered as very favourable, for they always constitute a dangerous complication to the principal affection. On ausculting her on the fifth day, we heard a fine crepitant rale, indicating the commencing of resolution in the lung; the tongue was less sticky; the sputa were still viscous, but were not streaked with red; the pulse had descended to 84 or 80; the heat was less burning; the skin more natural; and there was less anxiety about the patient. In fact, she appeared considerably better in several respects; although the night previous she had had slight delirium, which is a symptom of some gravity; and she also had passed her urine several times involuntarily, a circumstance to which, however, I did not attach much importance, although it is commonly regarded as very serious; for, in this patient, it only escaped during an access of cough. Still, we were not without uneasiness for the fate of the patient. The intestines having been the seat of irritation, and some traces still remaining, we were unable to resort to purgative medicines. On the next day, (Dec. 3,) there was no amelioration; a small dose of castor oil which was administered produced no effect; the belly continued somewhat tender and tympanitic; auscultation revealed well marked crepitation at the posterior part of the lung. The pulse was 88, and feeble; there was a state of great prostration.

Kermes Mineral was ordered, with an infusion of ground-ivy with soup, and a little wine. We employed these means to combat the extreme prostration of the patient. I may here call your attention to the importance of distinguishing between true debility and that state of weakness which is only apparent, which is called oppression of the forces, and which disappears under the use of an antiphlogistic treatment, more or less energetic. This species of weakness generally occurs at the commencement of severe inflammatory attacks, and is caused by the oppression of the vital forces by the inflammatory action. But when debility supervenes, when the disease has nearly ran its course, when the inflammatory symptoms have diminished, when the pulse has fallen, and is small, then, I say, this debility should not be confounded with the *expressio virium* of the ancients; it is genuine debility, and demands appropriate treatment. In this case there is that indication which the ancients called *vital*, that is to say, the indication to arrest life, which threatens to go—it is by the administration of stimulants and tonics with prudence that this result is to be obtained.

On December 6th she seemed better; her forces rallied; her pulse remained the same; where at first we had pure bronchial respiration, we now had nearly normal respiration, and we had every reason to anticipate a speedy resolution of the disease. From this time the patient continued to improve, and by the 18th of December was in a state of confirmed convalescence.

In our first examination of this patient, I insisted on the question, whether she had been exposed to any sudden change of temperature; and she answered in the negative. This circumstance seemed to me important, and I designedly was particular in my questions; for some physicians have insisted that pneumonia, as well as rheumatism, was always produced by exposure to cold. Now I have kept a register of a vast number of cases of pneumonia, and I pronounce this opinion unfounded. It no doubt happens, that in a large number of cases there is sudden exposure to cold sometime before the invasion of the disease; but it is, however, by no means the only cause, or, indeed, the chief cause. In such individuals, we should admit a predisposition which the exposure to cold has developed. The cold, in this circumstance, is only the occasion of the explosion of a preëxisting morbid state; in the same way, for example, that a simple indigestion can be the determining cause of a gastritis in a patient whose stomach was strongly disposed to inflammation: so that sudden exposure to cold has not all the importance in the production of pneumonia which some physicians would wish to accord to it.

Not so, however, of the initial chill; this is a phenomenon almost constant in pulmonary inflammation; it is in this affection that you most often observe it. Consequently, whenever the physician finds himself in presence of this symptom, he should immediately direct his attention towards the respiratory apparatus, and there he will almost always find some morbid phenomena in train of development; unless, indeed, other symptoms should not induce him to be arrested by some other organ. You should, therefore, never lose sight of the chill, at the debut, as an initial phenomenon of an acute inflammation of the lung. I do not deny that a chill may not occur at the commencement of other affections, as an enteritis, or a peritonitis, for example; but these are exceptions, and you should never base your diagnosis on exceptions. Hence, whenever I am called to a patient, and I find him with a well marked chill, if no other phenomena appertaining to other affections are present, I always look immediately to the lungs, for I know that most generally it will be the seat of the disease. I cannot tell you how often this sign alone has served me as a faithful guide in such cases; whilst other physicians, called to the same patient, and not taking this symptom into account, have imagined some other seat for the disease.

There is another symptom which merits your consideration—the pain in the side. In pleuro-pneumonia the seat of pain is most commonly in the mammary region, although the point of disease in the lung does not correspond, or extends much beyond it. An attempt has been made to explain the cause of this pain, and its want of correspondence with the seat of the disease, by saying that the visceral pleura exercises greater friction at the mammæ than at any other point of the thoracic parietes; pain manifests itself there when the serous membrane becomes inflamed. But if such were the case, the pain would not be limited to one circumscribed point, but would embrace all the surface, which is the seat of the friction of the visceral and costal pleuræ; it ought to shift about also, which never happens. The explanation of this phenomenon is, therefore, unknown; and we should not lose ourselves in vain hypothesis in attempting to account for it, but should be contented to remember the fact, which is one of great importance in diagnosis.

*Paris, Dec.* 1842.

